# Derivative chromosomes involving 5p large rearranged segments went unnoticed with the use of conventional cytogenetics

**DOI:** 10.1186/s13039-018-0374-4

**Published:** 2018-05-09

**Authors:** Emiy Yokoyama, Victoria Del Castillo, Silvia Sánchez, Sandra Ramos, Bertha Molina, Leda Torres, María José Navarro, Silvia Avila, José Luis Castrillo, Benilde García-De Teresa, Bárbara Asch, Sara Frías

**Affiliations:** 1 Departamento de Genética Humana, Instituto Nacional de Pediatría, Ciudad de México, México; 2 Laboratorio de Citogenética, Departamento de Genética Humana, Instituto Nacional de Pediatría, Ciudad de México, México; 3 Laboratorio GENETADI, Bilbao, Spain; 4 Laboratorio Diagen, Hospital ABC Santa Fe, Cuidad de México, México; 5 Departamento de Medicina Genómica y Toxicología Ambiental, Instituto de Investigaciones Biomédicas, Universidad Nacional Autónoma de México, Avenida IMAN no. 1, Torre de Investigación, Colonia Insurgentes Cuicuilco, Coyoacán, Ciudad de México Mexico

**Keywords:** Derivate chromosomes, Translocations, Intellectual disability, Congenital malformations, 5p deletion, 10p15 duplication, 9p22 duplication

## Abstract

**Background:**

In countries where comparative genomic hybridization arrays (aCGH) and next generation sequencing are not widely available due to accessibility and economic constraints, conventional 400–500-band karyotyping is the first-line choice for the etiological diagnosis of patients with congenital malformations and intellectual disability. Conventional karyotype analysis can rule out chromosomal alterations greater than 10 Mb. However, some large structural abnormalities, such as derivative chromosomes, may go undetected when the analysis is performed at less than a 550-band resolution and the size and banding pattern of the interchanged segments are similar. Derivatives frequently originate from inter-chromosomal exchanges and sometimes are inherited from a parent who carries a reciprocal translocation.

**Case presentation:**

We present two cases with derivative chromosomes involving a 9.1 Mb 5p deletion/14.8 Mb 10p duplication in the first patient and a 19.9 Mb 5p deletion/ 18.5 Mb 9p duplication in the second patient. These long chromosomal imbalances were ascertained by aCGH but not by conventional cytogenetics. Both patients presented with a deletion of the Cri du chat syndrome region and a duplication of another genomic region. Each patient had a unique clinical picture, and although they presented some features of Cri du chat syndrome, the phenotype did not conclusively point towards this diagnosis, although a chromosomopathy was suspected.

**Conclusions:**

These cases highlight the fundamental role of the clinical suspicion in guiding the approach for the etiological diagnosis of patients. Molecular cytogenetics techniques, such as aCGH, should be considered when the clinician suspects the presence of a chromosomal imbalance in spite of a normal karyotype.

## Background

In Mexico and other developing countries, the genetic approach for patients with intellectual disability (ID) and congenital malformations (CM) uses conventional G-banded karyotyping as the first-choice diagnostic test. It is usually performed at a 500-band level that allows the detection of 5–10 Mb abnormalities and results in an etiological diagnosis in approximately 3–10% of patients with a suspected chromosomopathy [1–3]. However, some structurally abnormal chromosomes with rearrangements larger than 5 Mb may go unnoticed by conventional cytogenetics. This may occur with derivative chromosomes, which are unbalanced intra- or inter-chromosomal rearrangements, in which the exchanged segments share a similar size and banding pattern, making them difficult to identify by conventional karyotyping [3, 4].

When a patient presents with a derivative chromosome, phenotypic evaluation and chromosome analysis of the parents are mandatory to rule out the presence of a balanced translocation in one of them [1]. In fact, 70% of derivatives are inherited and this information has a significant impact on genetic counseling [5, 6]. Comparative Genomic Hybridization arrays (aCGH) may uncover this type of abnormalities, because the finding of a combined deletion/duplication in the same patient points towards the presence of a derivative chromosome. Here, we describe the cytogenetic and clinical findings of two patients in whom a clinical phenotype consisting of ID and CM prompted the completion of aCGH despite having a normal 450-band karyotype. The two patients presented here, were ascertained through aCGH during the study of a cohort of 152 patients that presented with ID or CM and a normal conventional karyotype (manuscript in preparation).

Genomic DNA from the two patients and their parents was amplified and labeled using the CGH-labeling kit for oligo arrays (Enzo Life Sciences, New York, USA) and then analyzed with a 60 K oligonucleotide arrays according to the manufacturer’s protocol (Agilent, Santa Clara, USA). The slides were scanned using a microarray scanner with Surescan High Resolution Technology (Agilent, Santa Clara, USA). Image quantification, array quality control and aberration detection were performed using the Agilent Feature Extraction and DNA Analytics software (Agilent, Santa Clara, USA) according to the manufacturer’s instructions. Changes identified in the samples were visualized using the UCSC Genome Browser online tool (http://genome.ucsc.edu) and were compared to the Database of Genomic Variants (http://projects.tcag.ca/variation) to exclude copy number changes considered to be benign variants. The DECIPHER (Database of Chromosomal Imbalance and Phenotype in Humans using Ensembl Resources) (https://decipher.sanger.ac.uk/) and ECARUCA (European Cytogeneticists Association Register of Unbalanced Chromosome Aberrations) (http://umcecaruca01.extern.umcn.nl:8080/ecaruca/ecaruca.jsp) databases were used to assist with the genotype-phenotype correlation.

The rearrangements were validated using Fluorescence in situ hybridization (FISH) probes (Sure-FISH, Agilent, Santa Clara, USA). Molecular cytogenetic techniques, and GTG-banded karyotypes were performed on patients and their first-degree relatives to establish the origin (inherited or sporadic) of the chromosomal rearrangement and provide appropriate genetic counseling.

## Case presentation

Both patients were males, born to non-consanguineous parents. There was no family history of congenital diseases, intellectual disability, autism, seizures, neurological disorders, metabolic diseases, infertility or recurrent pregnancy loss. Physical examination revealed that both patients had weight, height and head circumference below the 5th percentile. An informed consent letter for each patient was obtained from the parents.

### Patient 1

**Fig. 1:**
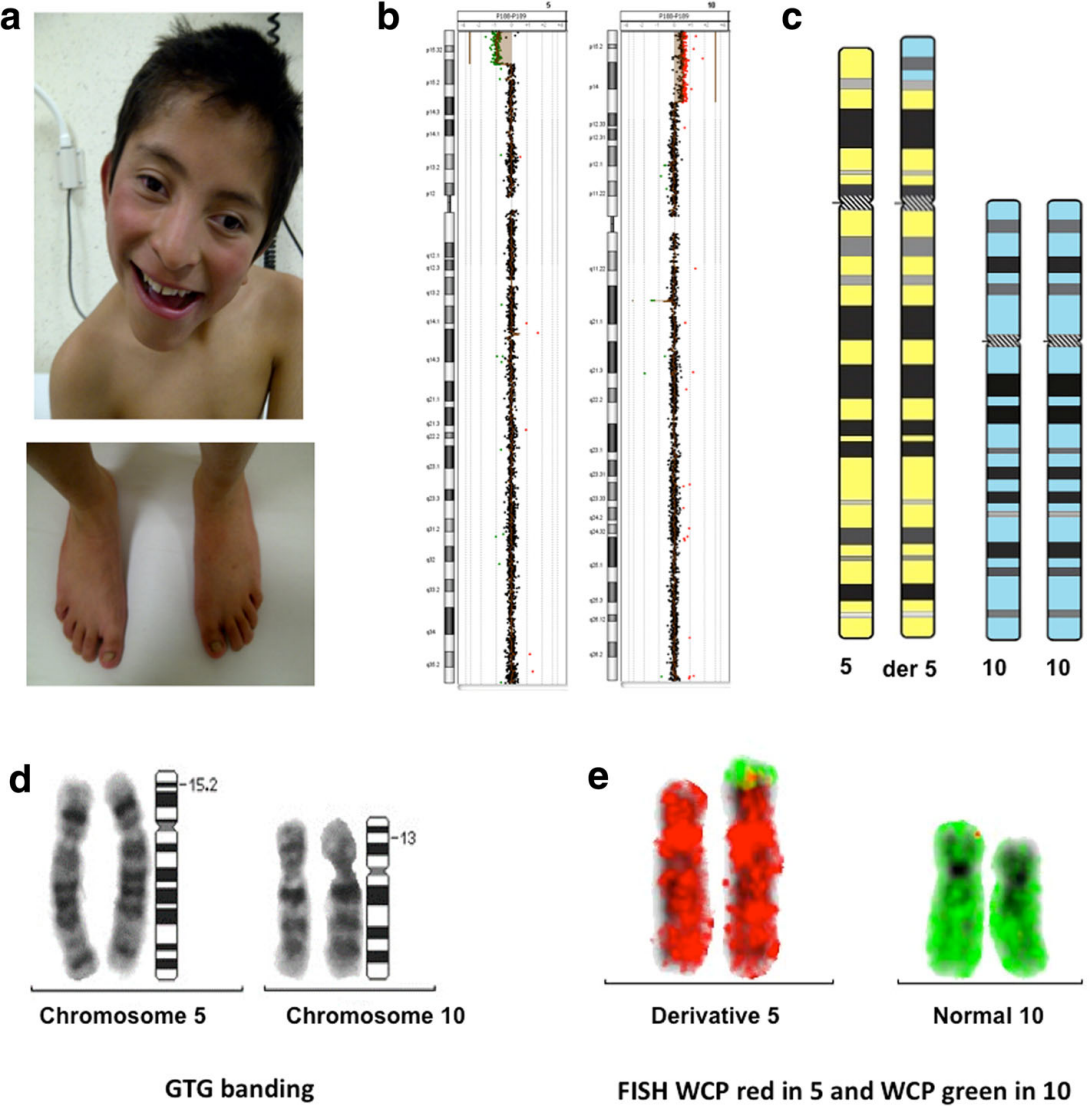
**a.** Long face, left eye strabismus, bilateral hallux valgus and hammertoes. **b.** Array CGH and FISH: 46,XY,der(5)t(5;10)(p15.2;p13)mat. ish der(5)t(5;10)(p15.2;p13)(wcp5+,wcp10+).arr[GRCh37/hg19] 5p15.33p15.2(151737_9215425)× 1, 10p15.3p13(148,206_14,869,993)× 3 mat. **c**. Ideogram with the two chromosomes involved (normal and translocated). **d**. GTG-banding of derivative chromosomes 5 and normal chromosome 10. Note the similar pattern of banding between the derivative chromosome 5 and the corresponding normal chromosome. **e**. FISH with WCP5 in red and WCP10 in green

Eight years old boy, born from the fourth pregnancy of a 33 years old mother and a 40 years old father; he has 3 healthy sisters. During pregnancy, decreased fetal movements were noted, and two ultrasound studies (USG) were reported as normal. He was delivered by cesarean section due to breech presentation at 37 weeks of gestation. He weighed 2800 g (between the 10th and 50th percentile), his height was 48 cm (50th percentile), and he received an Apgar score of 7/8. He required hospitalization for hypoglycemia and seizures. Delayed psychomotor development was noted at 9 months of age. Physical examination at the age of 3 years showed that the patient had posterior flattening of the skull, carp-shaped mouth (downturned corners of mouth), low-set ears, short neck, appendicular hypotonia, hands with prominent finger pads and multiple palmar creases, and a non-palpable right testicle. The computed tomography (CT) and magnetic resonance imaging (MRI) of the brain were normal. He also had brainstem auditory evoked potentials (BAEP), with bilateral severe hearing loss. USG of gonads reported both testes at the proximal third of the inguinal canal. Conventional karyotype at 450-band resolution was reported as normal, however, aCGH demonstrated the presence of a 9.1 Mb deletion of chromosome 5 and a 14.8 Mb duplication of chromosome 10 [Fig. 1a-e]. FISH analysis showed a normal pattern of the probes in the father’s sample, while a balanced translocation was observed in the mother and in two of his sisters. Following the 2016 International System for Human Cytogenomic Nomenclature (ISCN 2016) [7], the patient’s karyotype was 46,XY,der(5)t(5;10)(p15.2;p13)mat. ish der(5)t(5;10)(p15.2;p13)(wcp5+,wcp10+).arr[GRCh37/hg19]5p15.33p15.2(151737_9215425)× 1, 10p15.3p13(148,206_14,869,993)× 3 mat.

### Patient 2

**Fig. 2:**
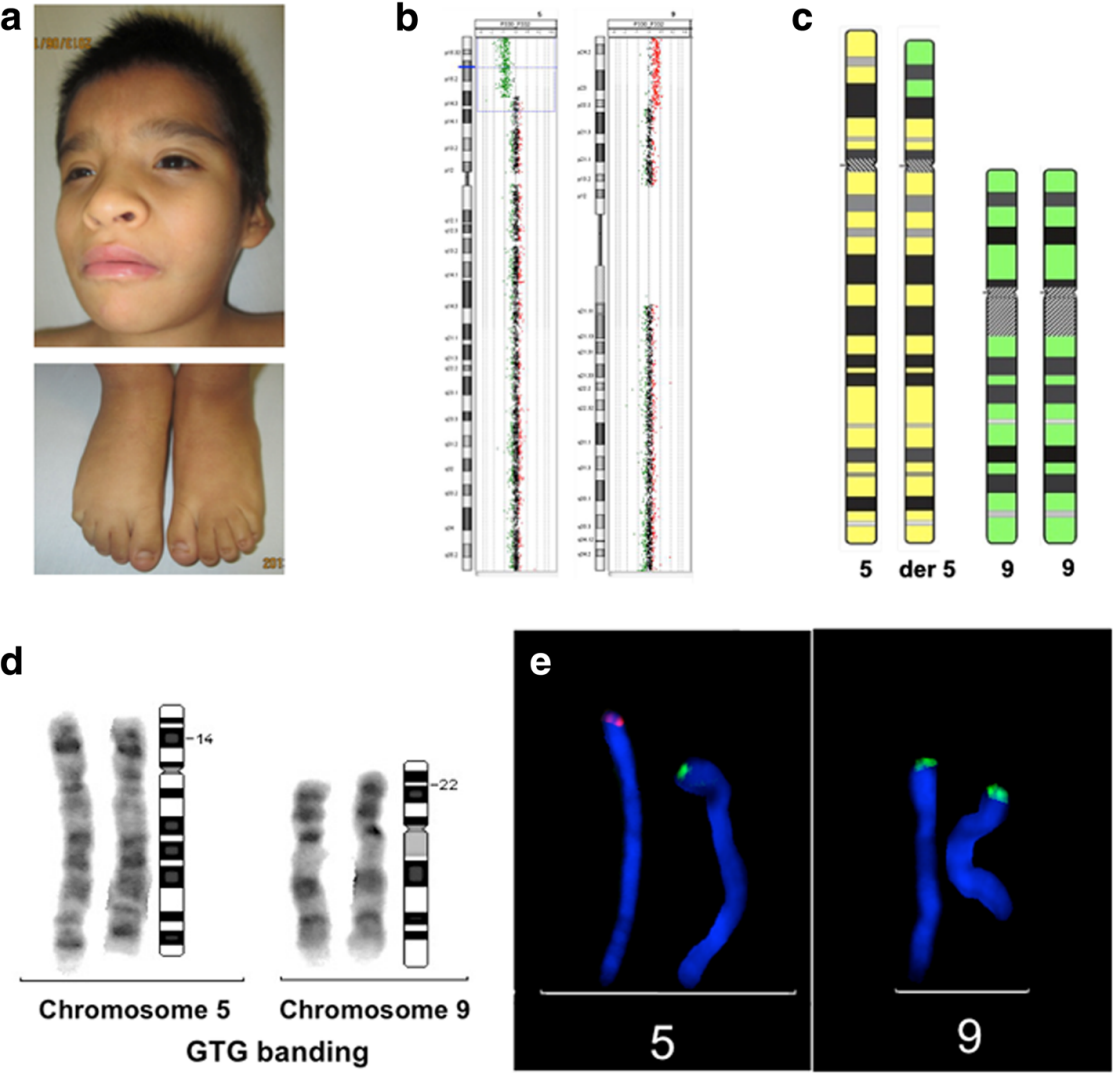
**a**. Long palpebral fissures, prominent antihelix, nail hypoplasia of toes and clinodactyly of the fifth finger. **b**. Array CGH with FISH: 46,XY,der(5)t(5;9)(p14.3;p22.1). ish der(5)t(5;9)(p14.3;p22.1)(PDCD6-,AHRR-,C5orf55-,EXOC3-,PP7080-,SLC9A3-,C9orf66+,DOCK8+,KANK1+).arr[GRCh37/hg19] 5p15.33p14.3(151737_20049770)× 1, 9p22.3p22.1(271,257_18,681,089)× 3 dn; **c**. Ideogram with the two chromosomes involved (normal and translocated); **d**. GTG-banding of chromosome 5 and 9 pairs; one of these chromosomes is the derivative 5 and the normal chromosome 9 from the patient, indicating the involved bands. **e**. FISH with probe located in 5p15.33 in red and probe located in 9p24.3 in green, showing the single dose of 5p and three doses of 9p

Seven years old boy, he is the first child of 30 years old parents. Two USGs were performed during the second trimester of pregnancy and intrauterine growth retardation was detected. Delivered at term by vaginal childbirth with a weight of 2175 g (below the 3th percentile). Birth height and Apgar score are unknown. He did not spontaneously breathe and required supplemental oxygen and hospitalization for 20 days. At the age of 8 months he was diagnosed with developmental delay. Physical examination revealed disproportion at the craniofacial-body level, round face, telecanthus, epicanthus, ears with posterior rotation, prominent helix and antihelix, short and wide philtrum, downturned corners of the mouth, micrognathia, heart murmur, abdomen with gastrostomy catheter, axial hypertonia, upper limbs with hypoplastic nails and bilateral clinodactyly of the 5th fingers, bilateral transversal palmar crease, and male genitalia but neither testis was palpable. Renal ultrasound reported left renal ectopia and crossed fused renal ectopia; BAEP indicated severe right hearing loss and moderate left hearing loss. Conventional GTG karyotype was reported to be normal but aCGH showed a 19.9 Mb deletion of chromosome 5 and an 18.5 Mb duplication of chromosome 9 [Fig. 2a-e]. The patient’s karyotype was defined following ISCN 2016 [7] as: 46,XY,der(5)t(5;9)(p14.3;p22.1). ish der(5)t(5;9)(p14.3;p22.1)(PDCD6-,AHRR-,C5orf55-,EXOC3-,PP7080-,SLC9A3-,C9orf66+,DOCK8+,KANK1+).arr[GRCh37/hg19]5p15.33p14.3(151737_20049770)× 1, 9p22.3p22.1(271,257_18,681,089)× 3 dn.

In Fig. 2d, it is evident that the derivative chromosome 5 shows a very similar G-band pattern than the normal chromosome 5, which explains why it was not detected in the conventional karyotype despite the large segment involved in the rearrangement. FISH with the specific probes did not show a balanced chromosomal rearrangement in either parent.

## Discussion

The prevalence of derivative chromosomes is unknown, although the estimated frequency of balanced reciprocal translocations ranges from 1 in 500 to 1 in 1000 in live births [4, 6]. The carriers of a balanced rearrangement may have decreased fertility, miscarriages and children with ID and CM. All of these are consequences of pregnancies with genomic imbalances due to the fertilization with a gamete that received the derivative chromosome during meiotic segregation [8–10].

### Genotype-phenotype correlation

We describe two patients with derivative chromosomes that despite having large chromosomal imbalances were not identified by conventional karyotyping. Furthermore, even though these rearrangements resulted in a 5p deletion, the patients did not present a phenotype in which the Cri-du-chat syndrome was readily recognized. The first patient’s karyotype is 46,XY,der(5)t(5;10)(p15.2;p13)mat carrying a chromosome 5 derivative that arose from a t(5;10)(p15.2;p13). The derivative chromosome results in a partial monosomy of 5p15.2 → pter, producing haploinsufficiency of the *SDHA* (5p15.33) and *SEMA5A* (5p15.31) genes that have been related to psychomotor retardation, microcephaly, pachygyria and microgyria. All these symptoms were present in our patient [11]. The der(5)t(5;10)(p15.2;p13) also results in partial chromosome 10 trisomy 10p13 → pter. This region contains the *AKR1C3* gene (10p15.1) that is involved in male gonadal development [12] although its overexpression is yet to be related to an altered function. Thus, it is possible that the cryptorchidism found in this patient is a phenotype due to the 5p deletion [13]. In addition, the *GATA3* gene (10p14) is involved in ear development, and abnormalities at this level have been linked to ear defects [14]. Our patient shares ID, growth delay, microcephaly, low-set ears, downturned corners of mouth, cryptorchidism and abnormal palmar creases with previously reported 5p deletion patients [13]. Patients with the 10p duplication present with ID, microcephaly, low-set ears, hearing impairment, cryptorchidism and abnormal palmar creases [15, 16].

The second patient also has a chromosome 5 derivative with a karyotype 46,XY,der(5)t(5;9)(p14.3;p22.1)dn leading to a partial monosomy of 5p14.3 → pter. This karyotype explains why this patient shared clinical manifestations with Cri du chat syndrome such as microcephaly, round face, epicanthus, telecanthus, downturned corners of mouth and dysplastic pinnae [17]. This derivative also results in partial trisomy of 9p22.1 → pter and the patient presents with clinical manifestations that have been previously reported in patients with a 9p duplication. These include ID, growth delay, microcephaly, hypertelorism, epicanthic folds, low-set ears, transverse fold, short stature, hearing loss, and nail hypoplastic [18, 19].

Cri du chat syndrome due to a translocation is rare (10–15%) [13] and the typical phenotype of this syndrome also depends on the extent and composition of the trisomic region involved. Both our patients have some clinical manifestations of Cri du chat syndrome, which was not initially suspected, because the phenotype was modified by the combination of the trisomic segments and the deletion. Therefore, we reviewed the literature and compared our patients’ phenotype with clinical manifestations of classical Cri du chat syndrome, and with those of dup(10)(p13p15.3) and dup(9)(p22.1p22.3) (Table 1). We found that, when the case is not detected neonatally, the classical phenotype is masked by the clinical manifestations of the trisomic regions, and the deletion of Cri du chat region is detected only when aCGH is done [20–23]. It is worth mentioning that we could not find other cases reported in the literature in which partial trisomies 10p13 → p15.3 or 9p22.1 → p22.3 coexisted with deletion 5p14.3 → pter or 5p15.2 → pter.

**Table 1:**
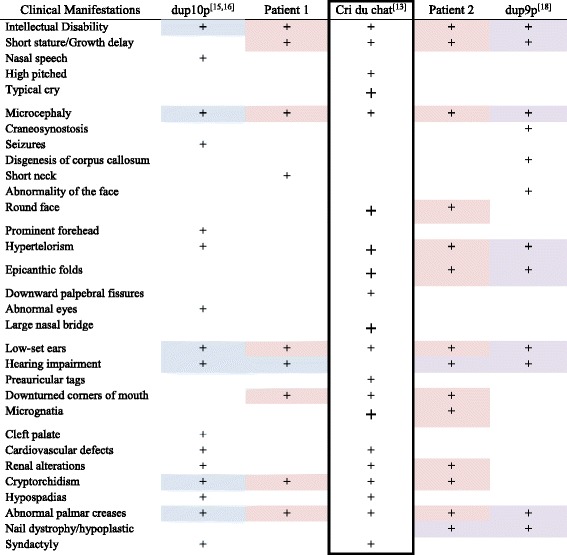
Comparison of the phenotype of patients 1 and 2 vs Cri du chat syndrome and clinical manifestations in patients with trisomy 10p or 9p

### Genetic counseling

**Fig. 3:**
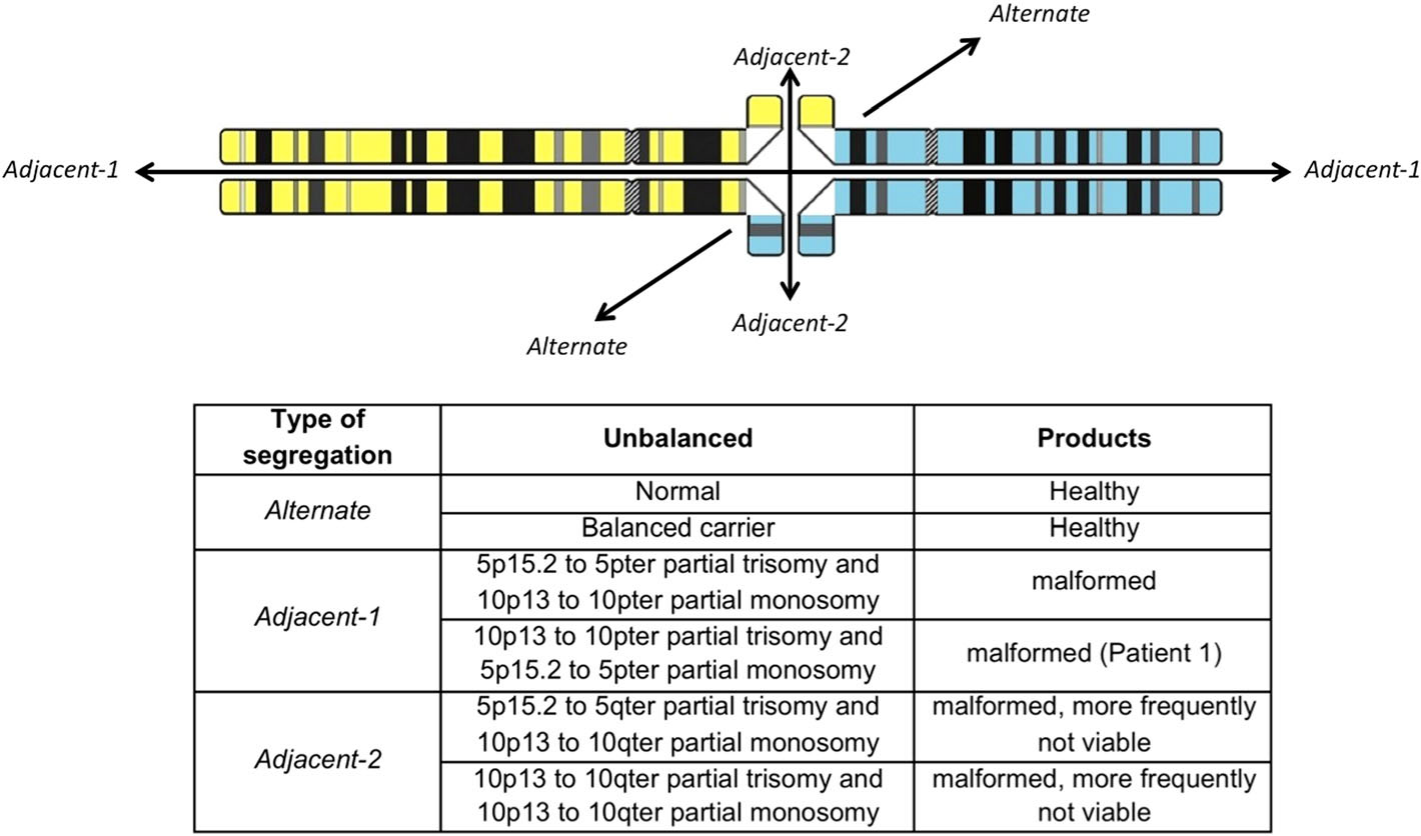
Pachytene cross between chromosomes 5 and 10. Possible gametes according to the type of segregation, as well as the expected manifestations and viability of the products

We were able to offer precise genetic counseling to these two families regarding the origin of these derivative chromosomes. It has been reported that 62% of rearrangements are paternal in origin, and only 38% are maternal [24]. However, when we analyzed the parents and siblings of patient 1 with specific FISH probes, we found that his mother and two of his sisters were carriers of a balanced translocation. This poses them at an increased risk of having offspring with ID and CM, as well as recurrent abortions, due to the generation of a gamete with an unbalanced rearrangement. In this family with identified carriers, knowing the breakpoints of the chromosomes involved in the rearrangement allowed us to build a pachytene cross (Fig. 3) and determine the percentages of possible normal, balanced, and unbalanced gametes. In addition to complement the medical history, it was possible to anticipate the viability of the unbalanced products and the products of the 2:2 segregation [8]. In this family, the theoretical risk of having a healthy child is 2 out of 6 (33%), with the healthy child receiving either the normal chromosomes or the balanced translocation (Fig. 3), and the empiric risk is 2 out of 4 (50%), considering only the viable products.

Genetic counseling for the second family is different because neither parent are carriers. Thus, the theoretical risk of recurrence is zero. However, we cannot rule out the presence of germline mosaicism in one of the parents or a non-paternity situation.

### The use of molecular cytogenetics methodology should be mandatory in patients with intellectual disability and multiple congenital malformations

Currently, it is well accepted that conventional karyotyping is an excellent and non-expensive methodology for detecting aneuploidies, low-level mosaicism and large -more than 10 Mb- rearrangements; however, detection of chromosomal structural abnormalities largely depends on the skills and experience of the cytogeneticist and yet, some alterations may go unnoticed [25]. To improve the detection of chromosomal abnormalities, molecular methodologies such as FISH or Multiplex Ligation-dependent Probe Amplification (MLPA) and chromosomal microarrays have been used [1]. Several groups have highlighted the economical and medical convenience of using microarrays as a first-line diagnostic test for detecting constitutional genomic imbalances [26–33]. The high cost of microarrays keeps these methodologies from being the first-line diagnostic tests in some developing countries, forcing a careful selection of patients in whom such studies are performed. Often, the few available microarrays are used in patients in whom a submicroscopic alteration is suspected, overlooking other patients who could also benefit from these types of analyses. Our cases support the benefit of offering microarray analysis to patients with a more severe phenotype such as ID and developmental delay, dysmorphic features or multiple CM, and when the clinical picture does not point toward a specific target region of the genome. A high rate of detection of unbalanced rearrangements using microarray methodology has been observed in patients with these referral indications [32]. It should be noted that microarrays can only detect unbalanced genomic regions, while the chromosomal locations of the duplicated or deleted regions material cannot be defined. In order to do so, the analysis must be complemented with the use of FISH to localize the segments in the karyotype.

## Conclusions

The phenotype of the patients with a derivative chromosome are unique, since it combines the clinical features of both the partial deletion and the partial duplication.

Noticeably, only when the clinical geneticist makes a detailed phenotype-genotype correlation, it becomes evident that the patient has clinical manifestations of the specific syndrome, such as Cri du chat syndrome in this study. The typical gestalt is modified by non-classical manifestations resulting from the new genome created by the chromosomal rearrangement, and the clinical diagnosis is not apparent.

Patients presented here are clear examples in which large chromosomal rearrangements go unnoticed by experienced cytogeneticists when using conventional cytogenetics. These cases highlight the importance of performing complementary analyses in patients with developmental delay or ID associated with CM using molecular cytogenetics techniques. The clinician’s suspicion of a chromosomal etiology for the patient’s condition, despite a normal conventional karyotype, is fundamental to support the need and demand the funding to perform further molecular cytogenetic testing that could positively impact the patient’s diagnosis, and to provide information regarding the biology of the disease.
